# Antibacterial Activity of the Green Synthesized Plasmonic Silver Nanoparticles with Crystalline Structure against Gram-Positive and Gram-Negative Bacteria

**DOI:** 10.3390/nano13081327

**Published:** 2023-04-10

**Authors:** Hemn Hassan Afandy, Dana Khdr Sabir, Shujahadeen B. Aziz

**Affiliations:** 1 Department of Physics, College of Science, Charmo University, Chamchamal 46023, Kurdistan Region, Iraq; 2 Department of Biology, Charmo Center for Research, Training and Consultancy, Charmo University, Chamchamal 46023, Kurdistan Region, Iraq; 3 Department of Medical Laboratory Sciences, College of Science, Charmo University, Chamchamal 46023, Kurdistan Region, Iraq; 4 Hameed Majid Advanced Polymeric Materials Research Laboratory, Physics Department, College of Science, University of Sulaimani, Qlyasan Street, Sulaymaniyah 46001, Kurdistan Regional, Iraq; 5 Development Center for Research and Training (DCRT), University of Human Development, Sulaymaniyah 46001, Kurdistan Regional, Iraq

**Keywords:** green tea, silver NPs, XRD, FIR, UV-vis, HR-TEM, antibacterial agents

## Abstract

Nanoparticles (NPs) have attracted considerable interest in numerous fields, including agriculture, medicine, the environment, and engineering. The use of green synthesis techniques that employ natural reducing agents to reduce metal ions and form NPs is of particular interest. This study investigates the use of green tea (GT) extract as a reducing agent for the synthesis of silver NPs (Ag NPs) with crystalline structure. Several analytical techniques, including UV-visible spectrophotometry, Fourier transform infrared (FTIR) spectroscopy, high-resolution transmission electron microscopy (HR-TEM), and X-ray diffraction (XRD), were used to characterize the synthesized Ag NPs. The results of UV-vis revealed that the biosynthesized Ag NPs exhibited an absorbance plasmonic resonance peak at 470 nm. According to FTIR analyses, the attachment of Ag NPs to polyphenolic compounds resulted in a decrease in intensity and band shifting. In addition, the XRD analysis confirmed the presence of sharp crystalline peaks associated with face-centered cubic Ag NPs. Moreover, HR-TEM revealed that the synthesized particles were spherical and 50 nm in size on average. The Ag NPs demonstrated promising antimicrobial activity against Gram-positive (GP) bacteria, *Brevibacterium luteolum* and *Staphylococcus aureus*, and Gram-negative (GN) bacteria, *Pseudomonas aeruginosa* and *Escherichia coli*, with a minimal inhibitory concentration (MIC) of 6.4 mg/mL for GN and 12.8 mg/mL for GP. Overall, these findings suggest that Ag NPs can be utilized as effective antimicrobial agents.

## 1. Introduction

Advances in the science of nanotechnology and using nanoscale materials with diverse applications in different fields have attracted scientists from different backgrounds to promote the field [1]. The use of NPs, acting as antimicrobial agents against multidrug-resistant (MDR) bacteria, is included [2,3,4]. From the World Health Organization (WHO) perspective, one of the greatest healthcare challenges of the twenty-first century is the MDR issue, and there are several healthcare infections that are difficult cure with currently available antibiotics [5]. For example, more than 35,000 people die annually in the United States from the infections caused by MDR bacteria [6]. Several approaches have been developed to tackle the MDR problem, such as utilizing metallic nanoparticles (NPs) as an antimicrobial agent (AMA) [7]. The AMA of the NPs relies on both the chemistry of the material and the physical properties of the NPs, for instance, shape, size, solubility, surface charge, and agglomeration. Recently, a tremendous focus has been placed on utilizing silver NPs (Ag NPs) as antimicrobial agents. This is due to their superior optical characteristics and strong antimicrobial action; in addition to the low cytogenic effect of NPs at low concentration [8], bacteria cannot develop resistance against NPs [9,10,11]. Ag NPs can inhibit the growth and kill microorganisms through attaching to the cell walls, altering the cell membrane permeability, and damaging the bacterial respiration process [12]. Additionally, a combination of Ag ions and the nitrogen-, oxygen-, and sulfur-containing genomic material of the cell can prevent DNA replication [9].

**Figure 1:**
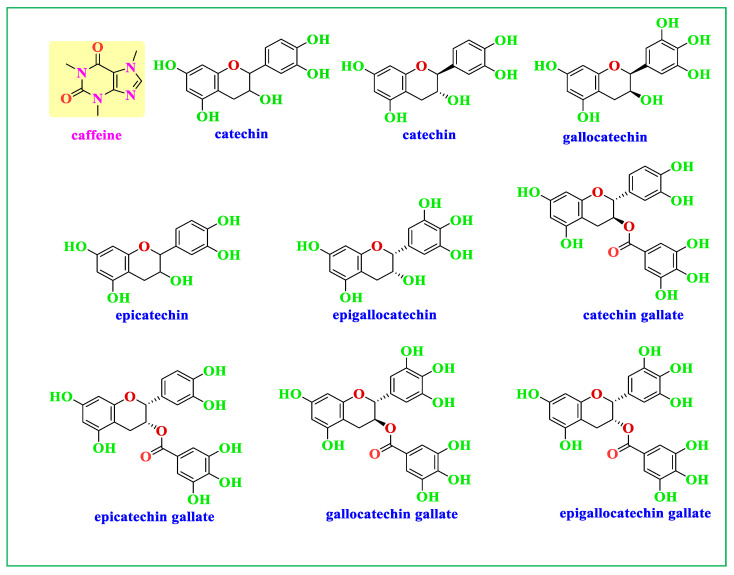
Components of GT extract enriched with caffeine and catechin (molecules that contain OH functional groups).

Green tea (GT) is a popular herbal drink that is consumed by people around the world. There are several significant ingredients in GT, and the most prevalent polyphenols (PPHLs) in GT are catechins, which are a class of flavonoid compounds and provide the antioxidant properties to the beverage. Epicatechin (EC), epicatechin-3-gallate (ECG), epigallocatechin (EGC), and epigallocatechin-3-gallate are the four primary catechins present in GT. Figure 1 displays the two most abundant components in GT, and the components of GT extract enriched by N, C=O, and OH functional groups, respectively. The amino acid theanine may help people feel peaceful and relaxed by having a calming impact on the brain. Other PPHLs found in green tea, including quercetin, kaempferol, and myricetin, have been associated with a number of health advantages [10]. Various methodologies have been applied in NP synthesis. The biosynthesizing of Ag NPs using plant metabolites as capping and reducing agents are often preferred. This is owing to the cost-effective, eco-friendly, and lower cytotoxicity effect on human cells [13,14,15,16,17]. In this study, the biosynthesis of Ag NPs using GT aqueous extract as a reducing agent is aimed, followed by assessing the activity against clinical isolates of both Gram-negative and Gram-positive bacteria.

## 2. Materials and Methods

### 2.1. Preparation of Ag NPs

**Figure 2:**
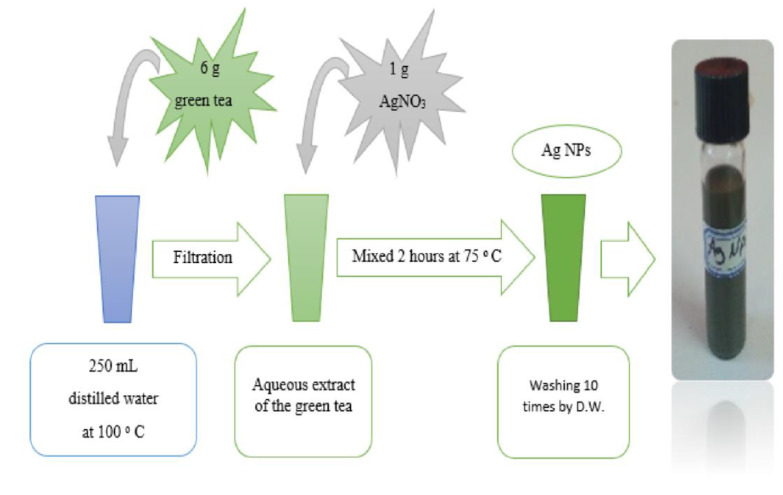
Schematic presentation of the Ag NPs preparation.

Aqueous extract of GT was used as a reducing agent to prepare Ag NPs. Figure 2 portrays the schematic fabrication of the Ag NPs. The procedure comprised weighing 6 g of GT powdered leaves and subsequently adding them to a 250 mL water bath (80 to 100 °C). The GT aqueous extract was used for the synthesis of Ag NPs. Afterwards, 1 g of silver nitrate (AgNO_3_) (>99.9%) was added into the GT extract, and the color of the mixture was changed from light to dark green. The mixture was then heated for two hours at 75 °C on a hotplate, followed by centrifuging the samples at 4600 rpm for 30 min. The Ag NPs were thoroughly washed ten times using sterile distilled water and the solution was kept at RT for later usage.

### 2.2. UV-Vis Spectroscopy 

Using a UV-Vis Spectrophotometric UV Line 9400 (SI Analytics, Düsseldorf, Germany) with a scanning capacity from 200 to 800 nm, we measured the UV-Vis absorption spectra of the prepared Ag NPs. For this purpose, 1 mL of liquid Ag NPs was stored in the UV-Vis cuvette.

### 2.3. FTIR Assay

For the dried essential Ag NPs in KBr pellets, a record of FTIR spectrum was carried out using a Perkin-Elmer FTIR spectrophotometer (Waltham, MA, USA), with a resolution of 2 cm^−1^ in a wavenumber range of 4000–400 cm^−1^. Herein, 100 mg of the KBr pellet was mixed with 5 mg of the sample. The FTIR spectrum was acquired for the dried pellet with the sample.

### 2.4. X-ray Diffraction (XRD)

An Empyrean Panalytical XRD diffraction meter (Empyrean XRD, Berlin, Germany) was used in recording the XRD at room temperature (RT). The operation electrical conditions of 40 kV and 45 mA were implemented during running. Other operating conditions comprised the use of a monochromatic X-ray radiation of a wavelength of k = 1.5406 A° at a glancing angle of 5B 2 h B 90, and a 0.05 step size during the scan of the samples.

### 2.5. High-Resolution Transmission Electron Microscopy (HR-TEM) Analysis

HR-TEM (Thermo Fisher Scientific, Hennigsdorf, Germany) was used to examine the surface morphology and size of silver NPs. The sample of the aqueous suspension of Ag NPs was prepared by dropping a little amount of the suspension onto the copper grids covered with carbon and letting the water evaporate at ambient temperature. On the basis of TEM micrographs, the size distribution of the resultant Ag NPs was observed. The crystalline structure was validated using HR-TEM images.

### 2.6. Bacterial Identification

Bacteria isolates used in this study were collected between January 2022 and March 2022 at the Harem Hospital/Sulaymaniyah, Iraq. For the identification of the isolates, a PCR machine was utilized for amplifying and sequencing the 16S rRNA gene. In the beginning, the colony PCR method was used to extract the bacterial genome [18], accompanied by the Taq Polymerase (2×) (GeNet Bio) to amplify the gene by means of the following primers: 7F: 5′- AGA GTT TGA TYM TGG CTC AG-3′ and 1501R: 5′- ACG GYT ACC TTG TTA CGA CTT-3′) [19]. The operation of PCR started with a denaturation process at 95 °C for 5 min, 30 cycles of denaturation for 30 sec at 95 °C, annealing for 30 sec at 60 °C, and an extension at 72 °C for 2 min. The final extension was performed at 72 °C for 5 min. The PCR products obtained from both forward and reverse primers were sequenced by Sanger DNA sequencing (Illumina, Macrogen-Republic of Korea) at Macrogen-Republic of Korea. The manual alignment and editing of the products were conducted using the Chromas program 1.0 (Technelysium, Macrogen-Republic of Korea) [20].

### 2.7. Construction of the Phylogenetic Trees

The phylogenetic trees were constructed with the ClustalX 2.1 and MEGA 7 programs (version 2.1, Dublin, Ireland). The sequence of the 16S rRNA gene of the isolates was aligned by ClustalX 2.1 with closely related sequences from the NCBI database. The MEGA7 program was employed to create the phylogenetic tree of the isolates with closely related bacteria using the neighbor-joining method (bootstrapped with 1000 replications) [21,22].

### 2.8. Antimicrobial Bioassay

The micro-dilution method was used to determine the MIC of the Ag NPs for the clinically isolated bacterial samples. To do this, a concentration range from 0.2 to 12.8 mg/mL of Ag NPs was applied. Initially, Mueller-Hinton broth (MHB) was used to grow the bacterial cells overnight at 35 °C to achieve 0.5 McFarland cells. The culture was then diluted by mixing 20 µL of it with 180 µL of MHB, and 20 µL of different Ag NP concentrations in the 96-well plates, and kept for 24 hrs at 37 °C. A plate reader (BioTek-ELx800) was used at 600 nm to measure the optical density (OD) of the bacterial growth and assess the impact of Ag NPs on the bacterial growth.

## 3. Results and Discussion

### 3.1. UV-Visible Study 

**Figure 3:**
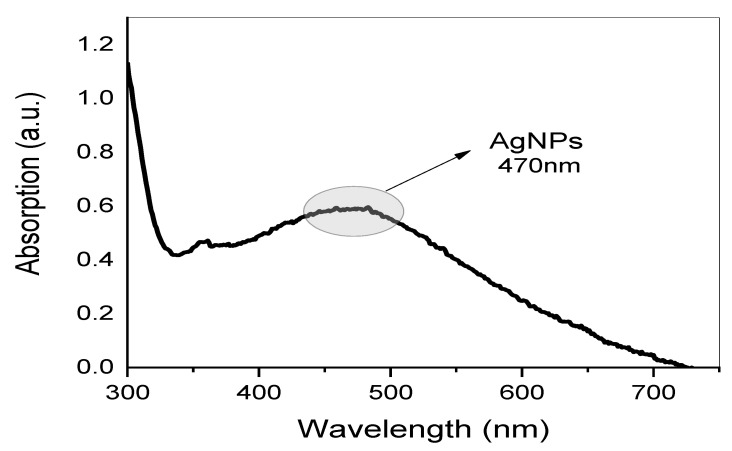
UV-visible spectrum of biosynthesized Ag NPs.

In the present study, Ag NPs were synthesized using aqueous GT extract as a reducing agent. A UV-vis spectrum of Ag NPs solution is displayed in Figure 3. By measuring UV-visible, the Ag NPs resonance peak can be identified in the 400–600 nm range, and thus its production can be ensured. Clearly, a broad absorption peak at 470 nm was observed in Figure 3, which verifies that the biosynthesized Ag NPs are in nano-range [23]. 

Metal clusters possess an absorption peak that is absent from the spectrum of bulk metals. This peak results from the collective oscillation of free-conducting electrons induced by the interaction of electromagnetic fields [24,25,26,27,28]. The conduction electrons in the metal are coupled to the electromagnetic field of the incident light, resulting in their oscillation and the creation of an electric field on the surface with limited depth of penetration [26,29,30]. On the right side of the main peaks, Figure 3 depicts no other broad peaks. Due to their symmetry, isolated silver spheres only exhibit a single plasmonic (PSN) resonance, as confirmed by theoretical models. Nonetheless, depending on the symmetry of the assembly, new resonances may emerge when they are arranged into small assemblies. Throughout the years, numerous applications based on the surface PSN resonance (PSNR) of Ag NPs have been proposed, especially in the fields of biosensing, surface-enhanced Raman scattering, and PSN circuitry [31]. It is logical to correlate the shape and size of the NPs to the majority of variations in surface PSN resonance (PSNR) locations. A PSNR (also known as plasmonic excitation) is ascribed to the conduction electrons’ collective oscillation in the valence band in response to an incoming beam [32,33]. There are many factors that can be counted that affect the location and intensity of localized PSNR bands, including the shape, concentration, behavior, and size of metal NPs [34,35]. The observation of PSNR peaks that are clearly broad, not sharp, can be ascribed to their small size and spherical shape [36,37]. Finally, since the PSNR peak only appears in metal NPs UV-vis spectra, the presence of this peak in the absorption spectrum is the most accurate indicator of NP creation [30]. More information about the shape of Ag NPs is provided in the later HR-TEM section.

### 3.2. Fourier-Transform Infrared Spectroscopy (FTIR)

**Figure 4:**
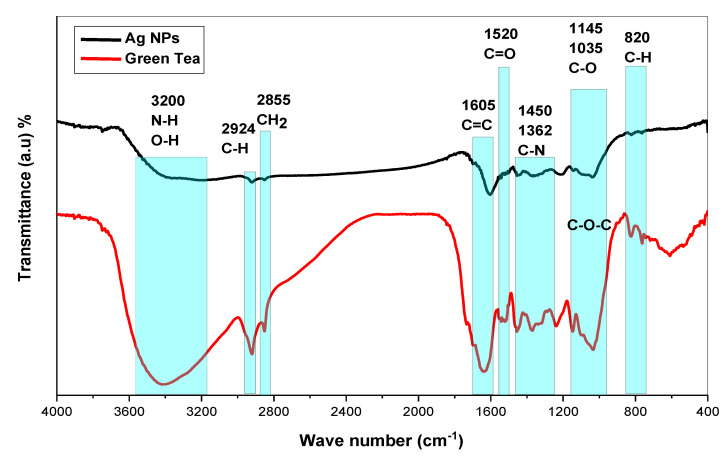
FTIR spectra for GT and biosynthesized Ag NPs and GT.

Investigation of Ag NPs and crude leaf extracts is helpful to confirm the appearance of several functional groups involved in the synthesis of Ag NPs. Figure 4 exhibits the FTIR spectra of the Ag NPs and the extract of GT together [26]. A large amount of organic chemicals are found in tea, for example, trace elements, minerals, volatile chemicals, proteins, glucides, amino acids, alkaloids, and the most intriguing substances, which are polyphenols (PPHLs) [38,39]. The core components of PPHLs were shown in Figure 1. There are also caffeine and theophylline along with PPHLs [40]. From Figure 4, it can be observed that all the peaks ascribed to GT also appeared in Ag NPs spectra, but with lower intensity and broader character. The adsorption and attachment of Ag NPs on functional groups of GT are responsible for lowering the intensity of the peaks and, consequently, broadening of peaks is expected.

The band identification at each wavenumber may be helpful to obtain deep knowledge about Ag reduction in GT solution media. Infrared spectra of GT and Ag NPs were observed to demonstrate bands at (3200 and 3410) cm^−1^ as a consequence of stretching vibrations in alcohol, N-H stretching in amines, phenols, and O-H groups in water [41]. In alkanes, the spectrum response near 2924 cm^−1^ is attributable to vibrations of symmetric C-H stretching [42,43]. The peak associated with wavenumber 2855 cm^−1^ is assigned to asymmetric stretching of the CH_2_ molecule [41]. Among aromatic rings, there is a strong band at (1636 and 1605) cm^−1^, while between 1525 and 1520 cm^−1^ in PPHLs, there is a strong band ascribed to C=O. There is an average C-N stretch at 1450 cm^−1^ in proteins, and a maximum stretch of 1370 cm^−1^ can also be assigned in proteins [44]. It is the amide band that accounts for the peak at 1236 cm^−1^ [45]. According to ref. [46], the peak at 1145 cm^−1^ associates to the C-O-C bridge anti-symmetric stretching. A band appears at 1035 cm^−1^ as a result of C-O stretching in amino acids [47]. The weak band at 825 cm^−1^ results from C-H out-of-plane bending. Finally, it is possible to decide that the chemical interactions among Ag NPs and functional groups are responsible for peak shifting and broadening in Ag NPs spectra [48]. It has been established that free amide groups allow protein molecules and Ag NPs to interact [49]. The protein’s amide linkage has a stronger ability to bond with silver, according to FTIR data, which could lead to the formation of a protein coating surrounding Ag NPs to avoid agglomeration and to stabilize the medium [50]. IR spectra indicate that GT and Ag NPs contain high concentrations of PPHLs [38]. According to the FTIR spectra of Ag NPs in Figure 4, the OH bending at 3410 cm^−1^ broadened and lost intensity. As shown in Figure 4, the C=O stretching of carboxylic acid groups moved from 1636 cm^−1^ to 1605 cm^−1^, indicating a decrease in their intensity. The fact that caffeine-related and other band vibrations are suppressed in the extract solution of tea leaves suggests that silver ions were collected by biomolecules and converted into Ag NPs. The formation of these organic capping agents may have resulted from the first reduction of silver ions through their complexation with the PPHL functional groups [51]. This is due to the fact that, when coordination between Ag^+^ cations, PPHLs, and caffeine occurs, the attachment of Ag^+^ cations causes a decrease in their vibrations, leading to an increase in their molecular weight or molecular mass. Advanced studies revealed that the main component of GT leaf extracts are PPHLs, which are enhanced by functional groups such as OH [52,53]. 

**Figure 5:**
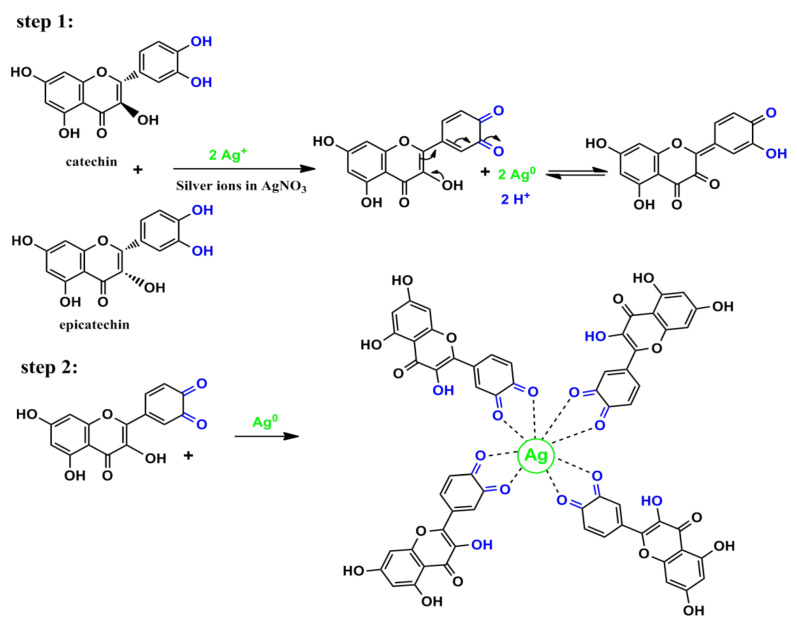
Proposed mechanism for the Ag NP production throughout silver ion–PPHL interactions.

A silver ion reduction map based on GT leaf extract PPHLs is presented in Figure 5 in light of previous [54,55,56] studies and the FTIR analysis of the current investigation. In a previous work, it was demonstrated using density functional theory (DFT) analysis that the catechol moiety of flavonoids in leaf extract solution is more stable than other flavonoid-OH groups [55]. Ag^+^ was able to form complexation with flavonoids and other PPHL groups, according to the hypothesized structure for the creation of the Ag complex (see Figure 5), so it is clear that electron transfer through oxidation from PPHLs to Ag^+^ is essential for the production of Ag NPs. The previous study found that the extract solutions of green leaves contained significant amounts of PPHL conjugates, PPHLs, carboxylic groups, hydroxyl groups, and conjugated double bonds [57]. It is worth mentioning that the oxidation of the PPHLs is accompanied by the reduction of Ag^+^ ions [58]. Specifically, the di-ortho-hydroxyl group in the B-ring facilitates the reduction process of the Ag^+^ ions to Ag NPs after releasing two electrons. It often occurs where Ag NPs are formed in the presence of flavonoids [56]. In addition to the strong chemistry relation between the electron-rich oxygen in the PPHLs and the silver orbital, the lone pair of electrons in PPHLs participate in the Ag NP dispersal and stabilization [58]. The Ag NP synthesis in the current report possesses similar FTIR spectra to those obtained by Kaur and Jaryal, [59] who prepared Ag NPs using biogenic tea waste. In order to acquire additional confirmation, a UV-Vis investigation of Ag NPs was carried out; the results are provided in the previous section. It was illustrated that Ag NPs demonstrate SPR band in the visible spectrum.

### 3.3. XRD and TEM Study

**Figure 6:**
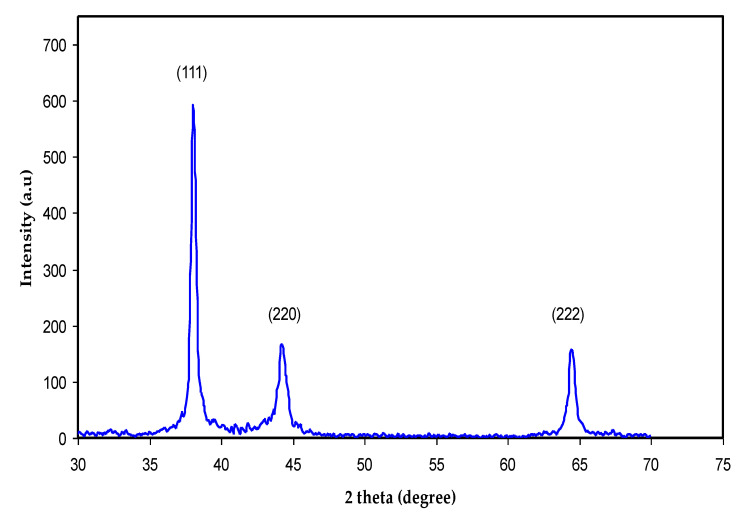
XRD pattern for biosynthesized Ag NPs at ambient temperature.

Crystalline and amorphous materials can be distinguished through XRD examination. Figure 6 shows the XRD pattern for biosynthesized Ag NPs at ambient temperature. In Figure 6, there are three sharp peaks at 2θ = 38°, 44°, and 64°, which correspond to silver crystals (111), (220), and (222), respectively. These results confirm that Ag NPs have a crystalline structure of FCC on the basis of the results published by other researchers [23,60]. The un-split (111) peak had the strongest peak intensity at 2θ = 38°, indicating that the (111) facet is the main Ag NP orientation, followed by the (220) and (222) facets [60,61]. According to the Debye-Scherrer equation, crystallite size (*D*) can be measured in nm.

(1) $$D=\frac{K\lambda }{\beta {}^{\circ}}\cos\left(\theta \right)$$

where *K*, λ, and β^0^ are the Scherrer constant, wavelength, and full width at half maximum (FWHM), and θ is the angle. Four parameters, including the *K* (0.95), the λ of X-ray (0.154 nm), the FWHM, and Bragg’s angle are obtainable. In Figure 6, from the width of the three peaks, it is possible to calculate the average crystallite size and measure it to be roughly 28.1 nm. Based on the Bragg diffraction law, this value is ascribed or equivalent to the distance of two planes of atoms. Thus, the particle size is about 14.05 nm. It is likely that Ag NPs with a diameter of below 16 nm are capable of being embedded into the cells of a different variety of bacteria, as well as directly interact with the membranes of these bacteria; thereby, poisoning of the cells occurs. Importantly, a large number of plant extracts can be used to prepare various NP sizes, shapes, and types. In particular, the most popular ones are plasmonic and metal oxide NPs [60,62,63]. Our XRD pattern agrees with those from earlier investigations. In this study, Ag NPs were shown to be cubically crystalline by diffract gram analysis, and a modest change in intensity may have been caused by the binding of biomolecules from the GT leaf extract to the surface of these NPs [23].

**Figure 7:**
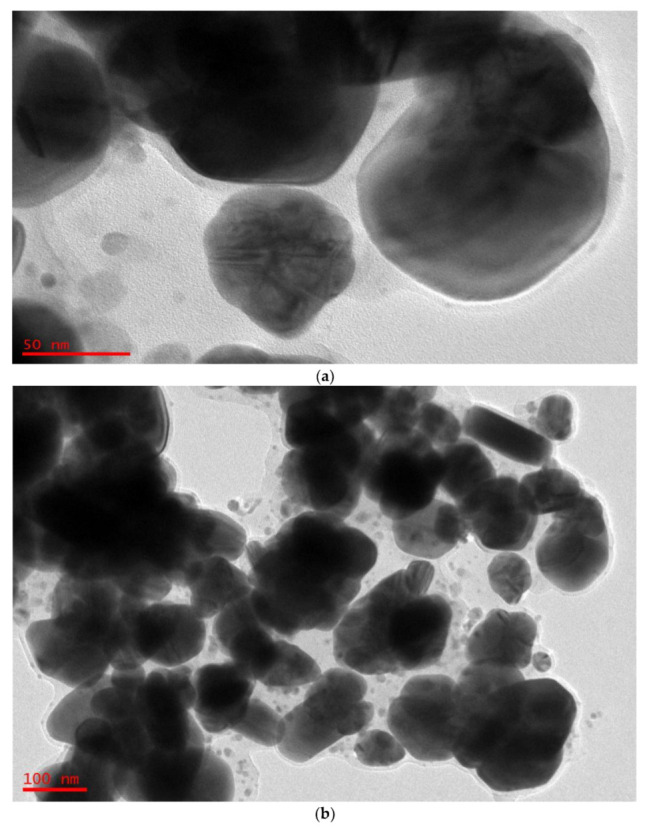

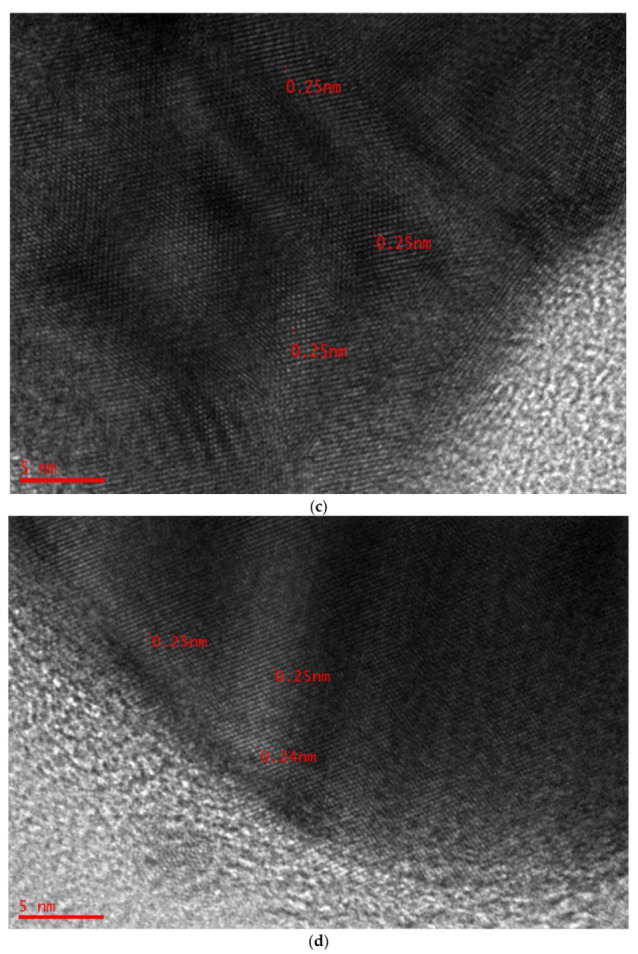
(**a**) TEM micrographs displaying spherical Ag NPs of 100 nm. (**b**) TEM micrographs displaying spherical Ag NPs of 50 nm. (**c**) HR-TEM image displaying IPDs of 0.25 nm. (**d**) HR-TEM image displaying IPDs of 0.24 nm.

The NPs are in fact silver crystals according to crystallographic (Figure 6) examination. Figure 7a,b shows a selection of TEM and HR-TEM images of the prepared Ag NPs. When the TEM micrographs were taken at a resolution of 100 nm, the particles were revealed as spherical in the 50 nm range. This HR-TEM image confirms that Ag NPs are crystalline, since there is an interplanar spacing of 0.25 nm (Figure 7C), which is caused by (111) planes of silver. These findings demonstrate that the NP population has an FCC form, which is very unique. The interplanar distances (IPDs) in the case of FCC NPs are quite close to 0.23–0.25 nm.

The XRD pattern and these results agree. They are connected to planes (111), (220), and (222), respectively, which make up a silver crystal’s FCC structure. The structure’s IPDs, however, are *d* = 2.40 nm, and they match plane (111) [64,65,66]. Previous research demonstrated that the IP lengths of 0.25 nm and 0.24 nm correspond to the crystallographic Miller indices (100) and (101), respectively [66]. The micrographs (see Figure 7c,d) show that the crystal symmetries of Ag NPs have face-centered cubic (FCC) dominancy based on the IPDs [64].

### 3.4. Bacterial Identification 

**Figure 8:**
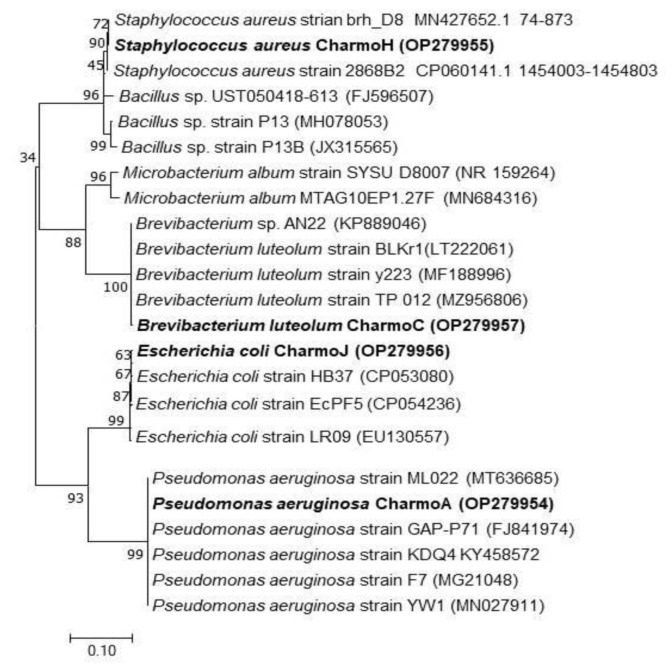
Phylogenetic sketch of *E. coli*, *P. aeruginosa*, *B. luteolum*, and *S. aureus* on the basis of the nucleotide sequence similarity of the 16S rRNA gene. The new isolates of the current study are denoted in bold. The access numbers of the 16S rRNA bacterial genes that were used to create the phylogenetic sketch are written in the brackets. The ClustalX and MEGA 7 programs, using the neighbor-joining method, bootstrapped with 1000 replicate runs, are used in creating the tree.

In the present work, four bacterial isolates were chosen to deal with the antibacterial activities of the biosynthesized Ag NPs. From the molecular identification that is based on the DNA sequence of the 16S rRNA gene, it was concluded that two of the isolates were GN bacteria: *E. coli* and *P. aeruginosa*. The other two isolates were GP and identified as *S. aureus* and *B. luteolum*. The partial 16S rRNA sequences of all four isolates were submitted to Genbank with the following strain names and accession numbers: *E. coli* strain J (Accession number: OP279956), *P. aeruginosa* strain A (Accession number: OP279954), *B. luteolum* strain C (Accession number: OP279957), and *S. aureus* strain H (Accession number: OP279955). The phylogenetic tree analysis of all four isolates shows (Figure 8) that each isolate is related to closely related species of bacteria in the database.

### 3.5. Antibacterial Susceptibility Testing

**Figure 9:**
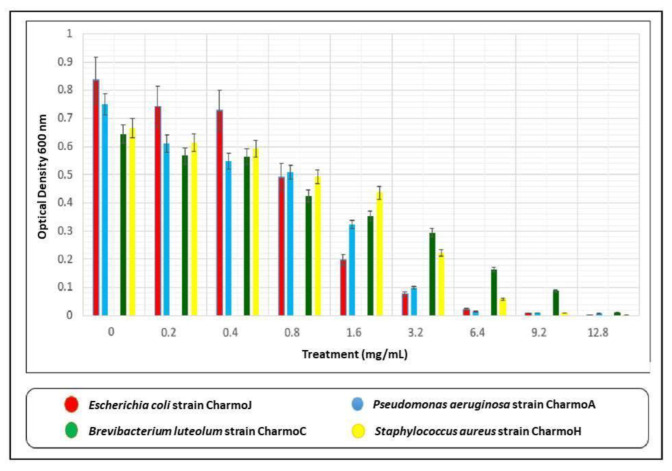
Effect of different concentrations of biologically synthesized Ag NPs on the growth of bacteria. Present data represent the mean values from three replicates of Ag NPs synthesis using GT aqueous extract.

Different bacterial species have shown different responses in dealing with biosynthesized Ag NPs [67]. In this study, Ag NPs at concentrations ranging between 0.2 mg/mL and 12.8 mg/mL were used. Figure 9 exhibits the effect of different concentrations of biologically synthesized Ag NPs on the growth of tested bacterial strains. 

The MICs of biosynthesized Ag NPs against the GP *B. luteolum* strain Charmo C and *S. aureus* strain Charmo H, and GN *P. aeruginosa* strain Charmo A and *E. coli* strain Charmo J were found, sequentially, at 6.4 mg/mL and 12.8 mg/mL, respectively. Previously, the AMA of the biosynthesized Ag NPs using GT extracts was demonstrated against different bacterial strains. Interestingly, the AMA of Ag NPs is greatly affected by the shapes and sizes of NPs [68,69,70]. There have been a few studies published recently on the antimicrobial activities of the biosynthesized Ag NPs [71,72,73]; however, the results of the current study are different in many ways in comparison to any of those previously published articles. Besides our novel approaches to preparing and characterizing the Ag NPs, such as FTIR, UV-vis, XRD, and Hr-TEM, advanced molecular and bacteriological techniques were used to accurately identify the studied pathogenic bacteria, such as PCR, which was used to amplify the 16SrRNA genes of the bacterial samples, DNA sequencing, and phylogenetic tree analysis, which is not used in any of the other published articles [71,72,73]. Although both [71,72] have demonstrated the antimicrobial activity of the biosynthesized Ag NPs, we also determined the minimal inhibitory concentration (MIC) of the synthesized NPs against the tested isolates—which is an important test that needs to be carried out to assess the toxicity of the potential therapeutic agents. Rolim et al. [73], on the other hand, determined the MIC of their Ag NPs against the tested bacteria, but the bacterial samples that were used in our study were locally isolated clinical samples, not ATCC samples. Chandra at al. [74] and Mobaraki et al. [75] also used Ag NPs, but not for antimicrobial activity. Perhaps the only part that is common between the previous studies and our current study is that green tea extract was used to biosynthesize Ag NPs. The novelty of the present work is the detailed characterization of the synthesized Ag NPs and the proposed method for Ag reduction in the GT extract medium. It has been documented that the spherical-shaped Ag NPs experience a stronger AMA against E. coli, S. aureus, and P. aeruginosa compared to the triangle shape [76]. Regarding the size of the NPs, the smaller NPs possess a stronger AMA [70]. For example, an average size of NPs of 5 mm has shown effective AMA of the Ag NPs used in this work [77].

It has previously been emphasized that the relatively small size of the spherical Ag NPs is appropriate for determining the decent AMA [62]. The production of reactive oxygen species (ROS) can destroy or at least harm the bacteria. Description of the detail (mechanism) of action and role of Ag NPs in preventing the growth of various bacterial species is crucial to have a comprehensive understanding of the antibacterial property of Ag NPs. Two distinct pathways of antibacterial action can be taken into consideration for limiting the growth of different bacterial species. Ag NP surfaces are transformed and stabilized in the first phase according to the bacterial environment through a number of processes, including disintegration, agglomeration, photochemical reaction, and release of Ag ions from Ag NPs. The second stage involves Ag NPs interacting with bacterial cell walls, which prevents bacterial proteins from doing their jobs and causes bacterial cell death by blocking bacterial growth. However, a variety of additional variables, including Ag NPs’ physical and chemical properties, size, shape, and ligand chemistry, as well as the medium, bacterial habitat, and concentration, also affect how effective they are at killing bacteria [78].

## 4. Conclusions

In conclusion, it has been shown that using GT extract to create Ag NPs is an efficient and promising way to create nanoparticles with antibacterial capacities. This work established that Ag NPs with crystalline structure can be produced using GT as a reducing agent. The synthesized Ag NPs were characterized via UV-vis, FTIR, high- HR-TEM, and XRD. The biosynthesized Ag NPs exhibited an absorbance peak at 470 nm, which reveals that most Ag NPs have a spherical shape. The FTIR analyses revealed band shifting and decrease in intensity of bands due to the attachment of Ag NPs to PPHL compounds. The XRD examination illustrates sharp crystalline peaks ascribed to face-centered cubic metallic Ag NPs. Additionally, HR-TEM showed that the synthesized particles were spherical with an average size of 50 nm. Gram-positive and Gram-negative bacteria were both strongly inhibited by the Ag NPs, with a minimum inhibitory dose of 6.4 mg/mL for GN and 12.8 mg/mL for GP. According to these findings, Ag NPs may be used as a powerful antibacterial agent across a range of industries, including agriculture, health, the environment, and engineering. Therefore, further investigation is required to maximize the production of Ag NPs, investigate their potential uses in agriculture and medicine, and reduce any possible damage to aquatic life. 

## Data Availability

Not applicable.
